# Work for the Wounded of the Allied Armies

**Published:** 1914-10-31

**Authors:** 


### HOSPITALS AND THE WAR.

(From our Special Correspondents.)

### Work for the Wounded of the Allied Armies.

### MILLER HOSPITAL, GREENWICH.

A lady, who earlier in the year endowed two beds at the Miller General Hospital for South-East London, has now generously furnished and equipped its empty ward so that the institution may do its appropriate share in the treatment of the wounded. The ward contains ten beds and the first batch, comprising that number of patients, arrived on October 16. The regiments represented are the 2nd King's Own Scottish Borderers; 1st Northamptonshire; R.F.A.; 2nd Leinsters; 1st Middlesex; 9th Lancers; 1st Loyal North Lancashire; 2nd Royal Sussex; and the Army Veterinary Corps. The men are mostly suffering from shrapnel wounds and are all making good progress.

The use of this ward in no way interferes with the hospital's work for the civilian population; in fact, in the other four wards in use there is room for a further fourteen beds beyond the limit of fifty-two imposed by King Edwards' Hospital Fund, but owing to lack of funds it has not yet been possible to open them officially.

### BATH: PHRASE-CARDS AND FRENCH-SPEAKING.

The first batch of wounded to arrive in Bath—some fifty Belgian soldiers—came in on Thursday, October 22, and reached the Great Western Railway Station at five o'clock, where the Mayor, Dr. Preston King, and a number of the men's and women's detachments of the St. John Ambulance Brigade, together with six nurses from the Royal United Hospital, with Doctors L. Beath and Mary Morris and an interpreter, were in waiting.

Immediately upon arrival bovril and one of Bath's specialities—Oliver biscuits—were served to the wounded in the train. They appeared to be in the best of spirits, and received a rousing reception from the crowd. Uniform was conspicuous by its absence. The transport work was quickly carried out under Assistant Commissioner Sprawson, of the St. John Ambulance Association, the wounded being conveyed in a number of private motor-cars. When the likelihood of wounded coming to Bath was known, a local garage owned by Messrs. Fuller constructed a four-stretcher motor-ambulance body, and within twenty-one hours had the vehicle ready for use. There were no stretcher cases, and only two required to be carried from the train.

Half of the wounded are being accommodated at the Royal United Hospital and the remainder at the Kingswood School Sanatorium, situated on Lansdown, which has kindly been placed at their disposal by Mr. W. P. Workman and the governors. This building, which is naturally adapted for the purpose, is capable of accommodating twenty-five patients—thirteen in a large ward on the first floor and nine in two smaller ones, while a small ward on the ground floor contains three beds, capable of being darkened for patients with eye affections.

Each bed has been provided with a "phrase-card" in French and English, by which patients will be enabled to make their wants known, and dressing-gowns, bed-socks, etc., have been provided by the local branch of the Red Cross Society. The cost of maintaining the wounded is, for the present at any rate, being borne by the school authorities. Dr. Leslie Beath is the medical officer, and the nursing arrangements are under the care of Miss Middleton, the school lady housekeeper, and formerly matron of Beverley Hospital, Yorks, who is assisted by a staff of one trained nurse and eight nurses from the Red Cross and St. John Ambulance Detachments. The soldiers were deeply impressed by the welcome they received, and those at the Royal United Hospital were much moved as the assistant secretary, Mr. H. Humphries, who speaks French, greeted each patient with the words, "We welcome you to our hospital, we welcome you to our city, we welcome you to our country, where you will be safe from the barbarians."

### BELGIANS AT BRISTOL.

A number of Belgian wounded were received at Cossham Hospital on Wednesday, October 21. Some of the wounded were accompanied by their wives and children, who were permitted to go to Cossham Hospital to see the wards, etc., where their men-folk are to be nursed, and were afterwards taken to special premises prepared for their reception.

### CARDIFF: 255 MORE CASES.

On Thursday afternoon, October 22, another contingent of wounded British soldiers, numbering 140, arrived from Southampton in a Great Central Railway Company's hospital train. In spite of rain and the fact that news of their coming was not general, a big crowd gathered. Local interest was intensified by the presence among them of many who belonged to the Royal Welsh Fusiliers. There was a bigger proportion of leg wounds than usual.

Forty were "lying-down" cases, but such disability did not prevent one of the men from holding his tobacco-tin firmly nor another from smoking a cigarette. As evidence of the quickness and efficiency with which soldiers are conveyed from the battlefield, a member of the R.A.M.C. stated that when at Dover recently men were brought over who only a day before had been fighting round Antwerp.

On the Sunday a further draft of British soldiers arrived, numbering 115, of whom only one was a "cot" case. The men manifested their usual stoicism, and several of them caused no little merriment by preferring hopping to the motor-car to being assisted. In the course of an interesting conversation an artilleryman said: "We have had some stiff work, I can tell you. Serious injuries? No; not a bit. A few have holes drilled through, but not serious! I have a 'scratch' on this arm—nothing big in it, but I suppose it's got to be attended to." Of these 255 cases, 90 went to Howard Gardens Hospital, 85 to Albany Road, 70 to Splott Road, and 10 to King Edward VII.'s Hospital. Of the 599 British and 235 Belgian soldiers admitted to the 3rd Western General Hospital, 137 and 176 respectively have been discharged or transferred to various branch hospitals. No deaths among overseas troops have occurred, but another member of a home defence unit has died of pneumonia. There are no men dangerously ill. Some idea of the valuable work which is being done by Colonel Hepburn, V.D., Major E. Maclean, M.D., and the staff in general can be gathered from the fact that from October 13 to October 25 some 600 cases have been admitted. It is heartening to see the effect of the fine organisation in every direction.

### FLAG DAY IN SOUTH WALES.

Cardiff has been the principal Welsh centre to which Belgian refugees have been sent by the London Reception Committee, and the arrangements made for the accommodation of as many as possible have been very elaborate.

October 22 was the Union Jack Day for the selling of Belgian, French, or British flags for the benefit of the refugees, in the organisation of which the Lord Mayor and Lady Mayoress, Dr. and Mrs. James Robinson, took great interest. The city and district were divided into fourteen wards, and hundreds of ladies were engaged in selling the flags and in obtaining gratifying prices for them. By eleven o'clock the supplies in many parts had been exhausted, and urgent requests for flags from headquarters and for ribbon from local shops were made with happily satisfactory results. A novelty was the touring of the district by an "auctioneering car." By a happy coincidence some 150 Belgians arrived in the afternoon. They were much cheered as they were driven in motor-cars to the pavilion at the Cardiff Arms Park, where a substantial meal had been provided for them.

### IPSWICH HOSPITAL AND ITS WAR WORKERS.

For a provincial hospital of 150 beds the part played by the East Suffolk and Ipswich Hospital in the present crisis cannot be said to be insignificant, and when one of its honorary staff was entrusted with the organisation and equipment of a complete unit for a new military hospital at St. Malo it was fitting that the authorities should name it "The Ipswich Ward."

Dr. J. Hossack, the honorary electro-therapeutist to the East Suffolk and Ipswich Hospital, had been invited by Sir Claude Macdonald to equip and take charge of a ward in a hospital on French soil, and within four days he left Ipswich with all his equipment, accompanied by fifteen nurses and a fully qualified hospital-trained sister as matron, who incidentally is a sister of the vice-chairman of the East Suffolk and Ipswich Hospital. All the dressings, bandages, etc., have been provided by Ipswich ladies. The work of this Ipswich surgeon in France has come under the review of the French Minister of War, who has visited the hospital at St. Malo and expressed himself pleased with its work.

A convoy of seventy-six wounded was sent to the East Suffolk and Ipswich Hospital last week, twenty-nine of the cases being transferred to "Broadwater," a private hospital equipped by Mr. Wm. Paul, one of the vice-presidents of the East Suffolk Hospital, now managed by the St. John's Voluntary Aid Detachment.

It is unfortunate that the hospital should lose its senior house-surgeon, Mr. Richard Charles, F.R.C.S.I., on the day following the arrival of the wounded. He has been with the hospital nearly two years, and was recognised as a skilful surgeon. However, he answered his country's call when the time came, and the board of management granted him six months' leave of absence. At the present moment five members of the honorary medical staff are serving their country either in foreign fields or at base hospitals in this country. The senior house surgeon is on the staff at Netley; four sisters (members of Queen Alexandra's Imperial Nursing Service) are doing duty with the troops. One of these, now in Belgium or North France, was the out-patient sister, who, besides her nursing qualifications, is a trained masseuse and a good linguist. Four nurses are members of the Territorial Nursing Force and are tending the wounded in military hospitals; and last, but not least, three porters are fighting for their country, one aboard ship and two on land. The rearrangement of the internal staff, coupled with the extra duties and multiplicity of forms and letters in connection with the wounded, is proving a considerable strain on the secretarial department, especially as all returns for the Broadwater private hospital pass through the secretary's hands.

### LEICESTER: AMBULANCE TRANSPORT.

The number of patients at the 5th Northern General Hospital is gradually diminishing. The death of a second soldier has been reported, full military honours being accorded at the funeral. There are now many vacant beds in readiness for further convoys of wounded should the need unfortunately arise. A welcome gift of 300 packs of playing cards by British playing-card manufacturers, through the Red Cross Society, is recorded.

An appeal is being made for funds for the cost of building and equipping a temporary chapel for the patients and staff for the accommodation of seventy persons, estimated at £200. The chapel was dedicated last Sunday by the Bishop of Peterborough, who was accompanied by the Bishop of Leicester, the Rev. W. Cyril Luxmoore, chaplain, and other clergy.

Good progress has been made in the appeal by the County Director, Voluntary Aid Detachment, Mr. A. W. Faire, for motor ambulances for the conveyance of wounded soldiers to the hospital. The contributions to the Mayor's Fund in connection with the National Relief Fund now amount to £23,000. The County Fund in connection with the same movement amounts to about £9,000, and the Leicester and Leicestershire Patriotic Fund totals £2,350. Arrangements have been made by the town and county authorities for the accommodation of 250 Belgian refugees. Further hospitality is urgently required.

An inquiry has been received by the authorities of the Leicester Royal Infirmary from the headquarters of the Northern Command as to the possibility of the institution receiving a convoy of 100 wounded soldiers direct from Southampton. At the quarterly meeting of the governors it was suggested that a member of the headquarters staff should inspect the hospital with a view to the discussion of the necessary arrangements. The local Voluntary Aid Detachment authorities have kindly undertaken the transport of the patients.

### LINCOLN: ARRIVALS FROM OSTEND.

The 4th Northern General Hospital (Territorial) is now completed and in full working order, with accommodation for 520 patients. It has been inspected by the Deputy Director of Medical Services, who expressed himself satisfied. The whole of the nursing staff has been housed in the new Nurses' Home and new ward of the Lincoln County Hospital, which buildings were fortunately sufficiently far advanced to enable them to be utilised.

Late at night on October 16, 216 wounded Belgian soldiers arrived in Lincoln, mostly from Ostend. All the ambulances attached to the hospital and a big fleet of motor-cars lent by Lincoln residents were waiting for them, together with a large detachment of R.A.M.C. officers and men. Within a little over an hour of their arrival at the station all were safely in bed at the hospital. Large batches of wounded soldiers, who were admitted some weeks ago and are now convalescent, have been transferred to Brocklesby Park, lent by the Earl of Yarborough, and to Petwood, Woodhall Spa, lent by Mr. and Mrs. Weigall.

### MONMOUTHSHIRE: ST. JOHN'S AMBULANCE SCHEME.

Splendid work is being performed by the St. John Ambulance members in this district. Ten centres, covering nearly the whole of South Wales and Monmouthshire, have been formed for the collection of clothing and money, and the central office in Cardiff has been open until 8.30 every night. £457 and some 5,000 articles have been received, the majority of the latter having been used to equip 278 men sent from South Wales to the military and naval hospitals in England and Ireland and to the general and stationary institutions abroad. The Association has already supplied 7,500 nursing and general duty orderlies for hospitals and the bearer companies. The Deputy Commissioner, Mr. Herbert Lewis, states that when leaving for France he took with him, in addition to articles of clothing, 23,000 cigarettes which had been collected from gentlemen in the streets of Cardiff for wounded soldiers at the Front, the gratitude of whom was unbounded.

In response to the Admiralty's call, fifty men have volunteered for the Sick Berth Reserve, and it is anticipated that the promised 200 will be obtained at an early date. Several gifts of money for motor ambulances to be sent overseas have been made, and it has been decided to form a motor corps, consisting of a captain, subaltern, two sergeants, a corporal, and two drivers for each car provided.

The Society has been asked to find the medical units for the Welsh Army Corps which is now being formed, and though this cannot be done at present, owing to the men they have enrolled for some years being required for other purposes, it is expected that some 450 of the recent enrolments will be handed over to the authorities to complete the above-mentioned corps' field ambulance.

### NETLEY: THE WELSH HOSPITAL.

The advance section, consisting of ten orderlies, under Sergeant-Major Chidzey, recently left Cardiff for Netley to complete the arrangements at the hospital. Prior to their departure they were inspected by Lieut.-Col. A. W. Sheen, the commanding officer and senior surgeon, whose appointment has been confirmed by the War Office. A week later Lieut.-Col. Sheen and the remainder of the staff departed. Each was presented with a gold badge with the words "The Welsh Hospital" inscribed upon it, the gift of Sir Wm. James Thomas. The institution is now ready for receiving patients, and sixty-four of its beds have been endowed.

### SHEFFIELD: MORE BEDS AND AMBULANCES REQUIRED.

The existing accommodation at Sheffield has already proved inadequate, although the base hospital, in addition to the extensive and admirably adapted training college placed at the disposal of the authorities by the Education Committee, includes a considerable number of beds both at the Sheffield Royal Hospital and the Royal Infirmary. Colonel Connell, commanding officer of the 3rd Northern General Hospital, has now reported to the Corporation and suggests that the Winter Street Hospital would be suitable for the purpose. In order that this request may be acceded to, arrangements are being made for the removal of the cases of consumption, for which this hospital has latterly been used, to the Corporation's other hospitals at Crimicar Lane and Commonside, where temporary buildings are being constructed. Further temporary structures are also being erected in the grounds of the Corporation's fever hospital at Lodge Moor—a few miles out of the city on the Derbyshire moors. The Winter Street Hospital was the first of the city's isolation institutions, and is situated in a fairly central and elevated position. The Corporation's five motor ambulances, which were built by and are now under the control of and manned by the trained ambulance section of the City Fire Brigade, with a couple belonging to the Sheffield Board of Guardians, have been placed at the disposal of the hospital authorities. For the less serious cases the Corporation motor-'buses are utilised. Even with all this assistance, however, Colonel Connell could do with more ambulances when, as was the case on October 23, a convoy arrived with, out of 140 patients, 40 stretcher cases. The R.A.M.C. men found valuable allies in the members of the Midland Railway Ambulance Corps, under Mr. H. T. Bromage. Most of these wounded men had been brought direct from the scene of operations outside Lille, without detention for other than the necessities of immediate attention in the field hospital, and some of the men had received their wounds within between two and three days of their arrival in Sheffield. The same day sixty of the Belgian soldiers who came to Sheffield a week before suffering from the effects of wounds were discharged from the base hospital as fit to return to the Front. A considerable number of Belgian wounded still remain here, however, and fraternise very happily with their British comrades, with a resulting rapid extension of the linguistic attainments of each.

### TORQUAY: THE ACCOMMODATION QUESTION.

Torquay was kept busy last week by the arrival of wounded soldiers. On Tuesday fifty-four convalescent non-commissioned officers and men were received at the Western Hospital for Consumption, which is not being used at present for its usual patients. The battles in which these men were wounded were the Aisne, Marne, and Meuse. One of the men told the writer that he fought in the battle from Mons right back to Paris, and that in the advance to the Meuse he had the bad luck to fall over a cliff, where his foot was injured. He also stated that his battalion lost in one battle all the officers except two, ninety men killed, and 100 missing.

On October 21 seventy wounded Belgian soldiers arrived. Forty-five were taken to the Torbay Hospital, five to Kent House Nursing Home, and the remainder to the "Oldway" American War Committee's Hospital at Paignton. One hundred and thirty English wounded were also received at Oldway Hospital during the week. The Belgian wounded were not serious cases; most of them could walk unaided. The men were dressed in various costumes, wore hats of every description, from the eighteenth-century beaver down to an ordinary cap. Some carried what was left of their boots on their backs, and were wearing felt slippers. Knapsacks made of what looked like raw cow-hide were strapped on their shoulders. Since their arrival many of them have been given new boots. On Saturday last, when several were taken to the Pavilion in tram-cars, they were as smart as an English regiment on parade. These men are eager to return to the Front.

On the same day fifty English wounded soldiers were admitted into the Torquay Town Hall temporary Red Cross Hospital (the first of the wounded received there). Twenty of them were cot cases.

Would it not have been better if these serious cases could have been sent to the Torbay Hospital, which is only 100 yards distant? Twelve additional wounded officers were received at "Stoodley Knowle," the private residence of Colonel C. R. Burn, M.P., which has been fitted up as a Red Cross hospital for wounded officers. On the occasion of Colonel Burn bringing dispatches from the Front last week, he came to Torquay and visited all the hospitals in the district.

### TUNBRIDGE WELLS: SHELL WOUNDS FROM ANTWERP.

After several false alarms and two nights of waiting the first batch of Belgian wounded soldiers arrived at Tunbridge Wells on a recent Wednesday morning, when some fifty men reached the town. They were all in a terrible condition, coming in many cases straight from Antwerp or other battlefields; several were suffering from burns, the result of explosions in the magazines of forts. Into the General Hospital twenty were admitted, the outpatient waiting hall being converted into a ward. Hot soup was given to each man in a few minutes after his admission. A few men with eye injuries were taken to the Eye and Ear Hospital and the remainder to West Hall, Chilston Road, which is a V.A.D. hospital under the local commandant, Miss Moore.

Two more English wounded were received into the General Hospital from Chatham on the same day, and a few more eye cases into the Eye and Ear Hospital also. The next day five men were discharged from the General Hospital to Chatham cured; they travelled to their destination as usual in private motor-cars, which returned with five fresh cases to fill the beds. The 16th again brought inquiries from the Chief Constable for beds for wounded Belgian soldiers, and eight more were promised by the General Hospital, the other institutions in the town offering their quota. All day they were expected, and finally word came through that at 11 p.m. the train would arrive; then the news came that 1.30 was the time, but in the end the poor fellows arrived at 5 a.m. The severe surgical cases were sent to the General Hospital, the eye cases to the Eye Hospital, the Homœopathic Hospital took six, and West Hall the remainder.

To accommodate the medical out-patients at the General Hospital use is being made of Trinity Parish Rooms, which are opposite the out-patient entrance, and have been kindly lent free of cost to the hospital by the authorities of the rooms. The surgical out-patients and casualties can be seen as usual at the hospital.

The War Office have written asking whether the committee of the General Hospital would allow their offer of beds for the sick and wounded troops to include members of the Territorial Force and of the new Army. The committee replied that their offer held good in respect of all branches of His Majesty's Forces, and whatever the hospital could do to help, with due regard to the claims of the civil population, should be gladly done.

**[A few accounts are unavoidably held over.]**

